# Risk of Ankylosing Spondylitis in Patients With Endometriosis: A Population-Based Retrospective Cohort Study

**DOI:** 10.3389/fimmu.2022.877942

**Published:** 2022-06-15

**Authors:** Zhihua Yin, Hui-Ying Low, Brian Shiian Chen, Kuo-Shu Huang, Yue Zhang, Yu-Hsun Wang, Zhizhong Ye, James Cheng-Chung Wei

**Affiliations:** ^1^ Institute of Rheumatology, Shenzhen Futian Hospital for Rheumatic Diseases, Shenzhen, China; ^2^ Institute of Biochemistry and Immunology, Chung Shan Medical University, Taichung, Taiwan; ^3^ School of Medicine, Chung Shan Medical University, Taichung, Taiwan; ^4^ Department of Applied Foreign Languages, Chung Shan Medical University, Taichung, Taiwan; ^5^ Department of Medical Research, Chung Shan Medical University Hospital, Taichung, Taiwan; ^6^ Division of Allergy, Immunology and Rheumatology, Chung Shan Medical University Hospital, Taichung, Taiwan; ^7^ Institute of Medicine, Chung Shan Medical University, Taichung, Taiwan; ^8^ Graduate Institute of Integrated Medicine, China Medical University, Taichung, Taiwan

**Keywords:** ankylosing spondylitis, autoimmune diseases, endometriosis, immunology, spine

## Abstract

**Objectives:**

Previous research has shown a possible relationship between endometriosis and autoimmune diseases. However, the relationship between endometriosis and ankylosing spondylitis (AS) is lacking. Therefore, we intended to find possible associations between endometriosis and AS using ICD-9 coding data in a population-based retrospective cohort study in Taiwan.

**Method:**

Data for this retrospective cohort study were collected from the Taiwan National Health Insurance Research Database (NHIRD) between 2000–2012. We collected 13,145 patients with endometriosis and a 78,870 non-endometriosis comparison cohort. Diagnoses of endometriosis and AS were defined by the International Classification of Diseases-9 (ICD-9-CM) code for at least 3 outpatients or 1 hospitalization. Propensity score matching by comorbidities, corticosteroids, and non-steroidal anti-inflammatory drugs (NSAIDs) usage were done for baseline comparability. Cox proportional hazard models were used to evaluate crude and adjusted hazard ratios.

**Results:**

The cumulative incidence of AS was higher in patients with endometriosis compared to the non-endometriosis comparison cohort (log-rank test, p = 0.015). The adjusted hazard ratio (aHR) of incidental AS in patients with endometriosis was 1.61 (95% CI = 1.11 to 2.35) in comparison to the non-endometriosis comparison cohort. An increased risk of AS was also observed in subjects with major depressive disorder (aHR = 5.05, 95% CI = 1.85 to 13.78). Stratified analyses of age subgroups showed consistent results. NSAID users had a lower risk of AS than NSAID non-users (aHR 4.57 vs 1.35, p for interaction = 0.031).

**Conclusions:**

In this retrospective population-based cohort study, we found a higher risk of AS in patients with endometriosis. We suggest that clinicians should pay attention to the occurrence of AS in patients with endometriosis.

## Introduction

Endometriosis is a common benign gynecologic disease with active endometrial cells outside the uterine cavity. Epidemiological studies show that the prevalence rate is about 10–15% of women within the reproductive age bracket worldwide (1). The pathogenesis of endometriosis has not been completely elucidated. Studies have shown that the immune system plays an important role in the pathogenesis of endometriosis (2). The imbalance of the immune system is associated with invasion, proliferation, and angiogenesis of the endometrium. In terms of endometriosis patients, the elevated number of macrophages and increased level of tumor necrosis factor (TNF)-α could provide an inflammatory environment for endometriosis progress; the decreased cytotoxicity of natural killer (NK) cells and elevated concentration of regulatory T cells could decrease immune surveillance and then promote the establishment of endometriotic lesions. Furthermore, Th17 cell subset may also play a role in endometriosis.

A recent meta-analysis has summarized the association between endometriosis and autoimmune diseases from several previous studies (3). Some studies have demonstrated that patients with endometriosis have a higher incidence of autoimmune diseases, namely, rheumatoid arthritis (RA), systemic lupus erythematosus (SLE), Sjögren’s syndrome (SS), and multiple sclerosis (MS). However, the association between endometriosis and ankylosing spondylitis (AS) has never been reported. Endometriosis and AS share the same immunologic characteristics such as TNF-α and Th17 pathway. Therefore, in this study, we designed a retrospective cohort study to investigate the risk of AS in endometriosis women.

## methods

### Data Source

The research was a retrospective cohort study based on the National Health Insurance Research Database (NHIRD), which enrolled almost 99% of the population of 23 million beneficiaries in Taiwan. The database included all insurance claims data, including outpatient visits, emergency room visits, and hospitalizations. One million subjects were sampled from the 23 million beneficiaries, and the data were collected from 1999 to 2013. The sampled database was deidentified, and the study was approved by the Institutional Review Board of Chung Shan Medical University Hospital.

### Exposure and Patient Selection

The study cohort included newly diagnosed female endometriosis from 2000 to 2012 with ICD-9-CM coding of 617 for outpatient visits ≥3 times or hospitalization ≥1 time in the NHIRD dataset. The index date was set as the date of the first endometriosis diagnosis for the patient. To ensure a new-onset subject, we excluded the diagnosis of ankylosing spondylitis (ICD-9-CM = 720.0) before 2000. The non-endometriosis comparison group was defined with participants who had never been diagnosed with endometriosis from 1999 to 2013.

### Outcome

The outcome was the incidence of ankylosing spondylitis, defined by ICD-9-CM coding of 720.0 for at least three outpatient visits or one hospitalization after the index date of endometriosis. The study was followed until the occurrence of ankylosing spondylitis, or 31 December 2013, or withdrawal from the national insurance system, whichever occurred first.

### Covariates

The baseline characteristics were matched by age, sex, and co-morbidities, namely, hypertension (ICD-9-CM = 401–405), hyperlipidemia (ICD-9-CM = 272.0–272.4), chronic liver disease (ICD-9-CM = 571), major depressive disorder (ICD-9-CM = 296.2, 296.3), chronic kidney disease (ICD-9-CM = 585), chronic obstructive pulmonary disease (ICD-9-CM = 490–492, 494, 496), diabetes (ICD-9-CM = 250), coronary artery disease (ICD-9-CM codes = 410–414), cerebrovascular disease (ICD-9-CM codes = 430–438), and cancer (ICD-9-CM = 140–208). Those comorbidities were defined before the index date within one year and at least three outpatient visits or once hospitalized. Additionally, corticosteroids and non-steroidal anti-inflammatory drugs (NSAIDs) usage, defined by a prescription for at least 30 days within the first year, was calculated.

### Matching

**Figure 1:**
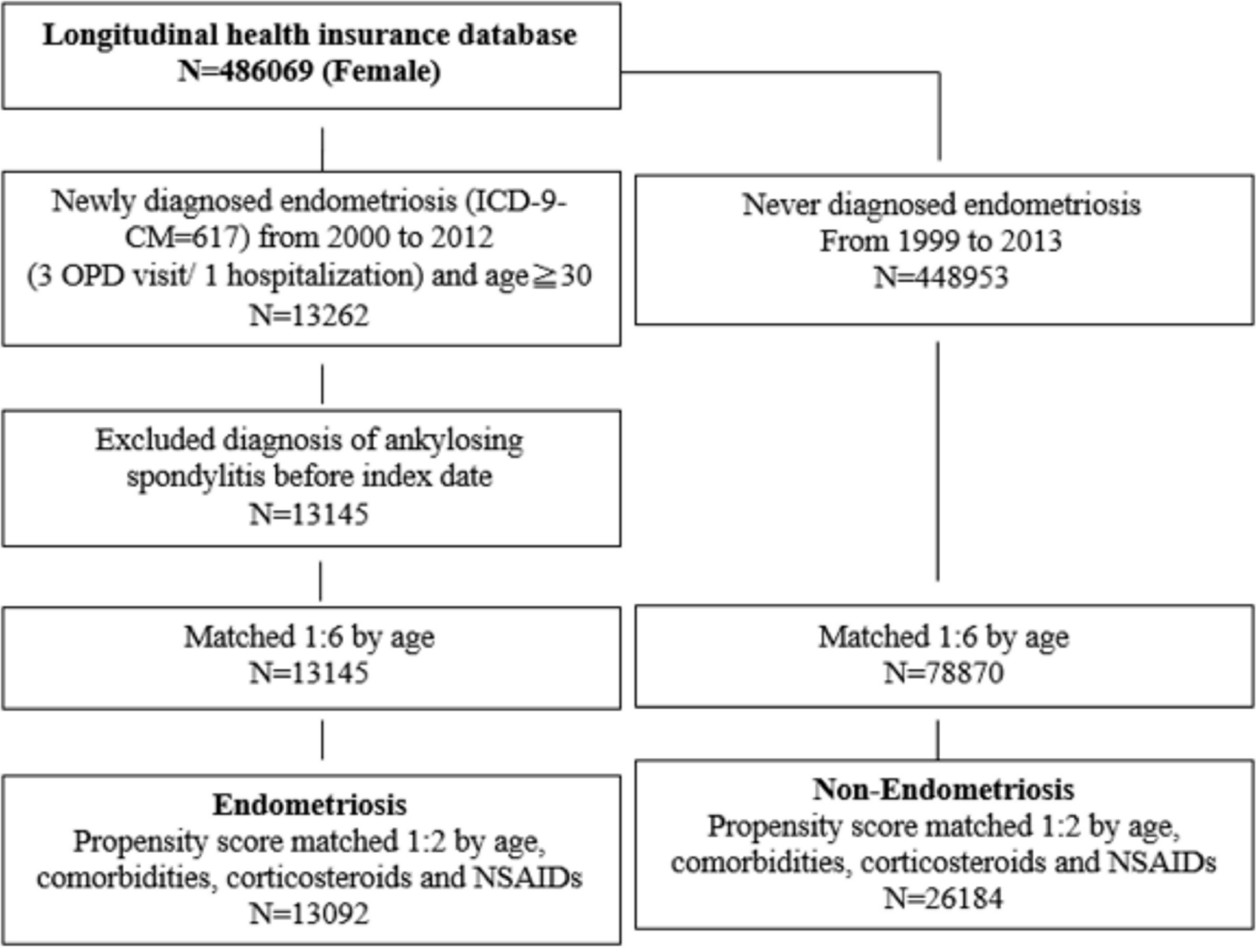
The enrolling criteria of this study.

As shown in **Figure 1**, 1:6 matching by age and index date was performed between both groups. Then, 1:2 propensity score matching was performed on comorbidities, corticosteroids, and NSAID usage between the two groups to ensure baseline comparability. The propensity score was a probability that was estimated through logistic regression. The binary variables were the endometriosis and non-endometriosis groups. By matching the propensity score, it could balance the heterogeneity between two groups.

### Statistical Analyses

To compare the characteristics of the endometriosis and non-endometriosis groups, the Chi-square test for categorical variables and the Student’s t-test for continuous variables were used. The Kaplan–Meier analysis was used to calculate the cumulative incidence of AS and the log-rank test was used to test the significance. A Cox proportional hazard model was used to estimate the hazard ratio of AS between the endometriosis and non-endometriosis groups, and it was adjusted for age, comorbidities, corticosteroids, and NSAIDs. We made the proportional hazard assumption. The p-value of the time-dependent covariates was 0.771, which satisfied the proportional hazards assumption. The statistical software being used in this research was SPSS version 18.0 (SPSS Inc., Chicago, IL, USA).

### Patient and Public Involvement

This research was conducted without participant involvement. The participants of the retrospective cohort study were not invited to comment on the study design and were not consulted to interpret the results. Participants were not invited to contribute to the writing or editing of this document for readability or accuracy.

## Results

### Analysis of Comorbidities and Medications in Endometriosis Patients

The characteristics of the enrolled 13,145 patients with endometriosis and 78,870 age-matched comparison cohorts are summarized in **Table 1**. Compared with the comparison cohort, patients with endometriosis were more likely to have the comorbidities, namely, hypertension (6.3% vs. 4.5%), hyperlipidemia (2.5% vs. 1.8%), chronic liver disease (2.3% vs. 1.4%), major depressive disorder (0.9% vs. 0.5%), COPD (1.2% vs. 0.8%), diabetes (2.7% vs. 2.0%), coronary artery disease (1.3% vs. 0.7%), cerebrovascular disease (0.6% vs. 0.4%), and cancer (2.7% vs. 1.1%), except for chronic kidney disease (0.2% vs. 0.3%). Consistent with the comorbidity analysis, most medications for these comorbidities, including costicosteroids (59.3% vs. 43.6%) and NSAIDs (77.6 vs. 58.8%), were significantly more frequently used in the endometriosis group than in the comparison cohort group.

**Table 1:** Demographic characteristics of enrolled endometriosis and non-endometriosis subjects.

	Before PS^1^ matched					After PS^1^ matched				
	Endometriosis (N = 13,145)		Non-Endometriosis (N = 78,870)			Endometriosis (N = 13,092)		Non-Endometriosis (N = 26,184)		
	n	%	N	%	p-value^2^	n	%	N	%	p-value^2^
Age (years)					>0.999					0.967
30–39	5,414	41.2	32,484	41.2		5,395	41.2	10,784	41.2	
40–49	6,511	49.5	39,066	49.5		6,500	49.6	13,025	49.7	
≧50	1,220	9.3	7,320	9.3		1,197	9.1	2,375	9.1	
Mean ± SD^3^	41.7 ± 6.9		41.7 ± 6.9		>0.999	41.7 ± 6.9		41.7 ± 6.9		0.307
Hypertension	828	6.3	3,557	4.5	**<0.001**	806	6.2	1,608	6.1	0.953
Hyperlipidemia	335	2.5	1,403	1.8	**<0.001**	328	2.5	635	2.4	0.628
Chronic liver disease	304	2.3	1,083	1.4	**<0.001**	296	2.3	597	2.3	0.905
Major depressive disorder	115	0.9	410	0.5	**<0.001**	113	0.9	249	1.0	0.390
Chronic kidney disease	23	0.2	210	0.3	0.054	23	0.2	46	0.2	>0.999
COPD^4^	164	1.2	621	0.8	**<0.001**	160	1.2	314	1.2	0.845
Diabetes	360	2.7	1593	2.0	**<0.001**	353	2.7	653	2.5	0.231
Coronary artery disease	171	1.3	589	0.7	**<0.001**	168	1.3	310	1.2	0.398
Cerebrovascular disease	76	0.6	340	0.4	**0.020**	76	0.6	137	0.5	0.466
Cancer	354	2.7	835	1.1	**<0.001**	302	2.3	579	2.2	0.547
Corticosteroids	7,792	59.3	34,421	43.6	**<0.001**	7,740	59.1	15,487	59.1	0.959
NSAIDs^5^	10,196	77.6	46,378	58.8	**<0.001**	10,143	77.5	20,309	77.6	0.844

^1^PS, Propensity score.

^2^p-value was calculated by Chi-squared test.

^3^SD, standard deviation.

^4^COPD, chronic obstructive pulmonary disease.

^5^NSAIDs, non-steroidal anti-inflammatory drugs. The bold values stand for statistical significance.

### Cumulative Risk for Ankylosing Spondylitis

**Figure 2:**
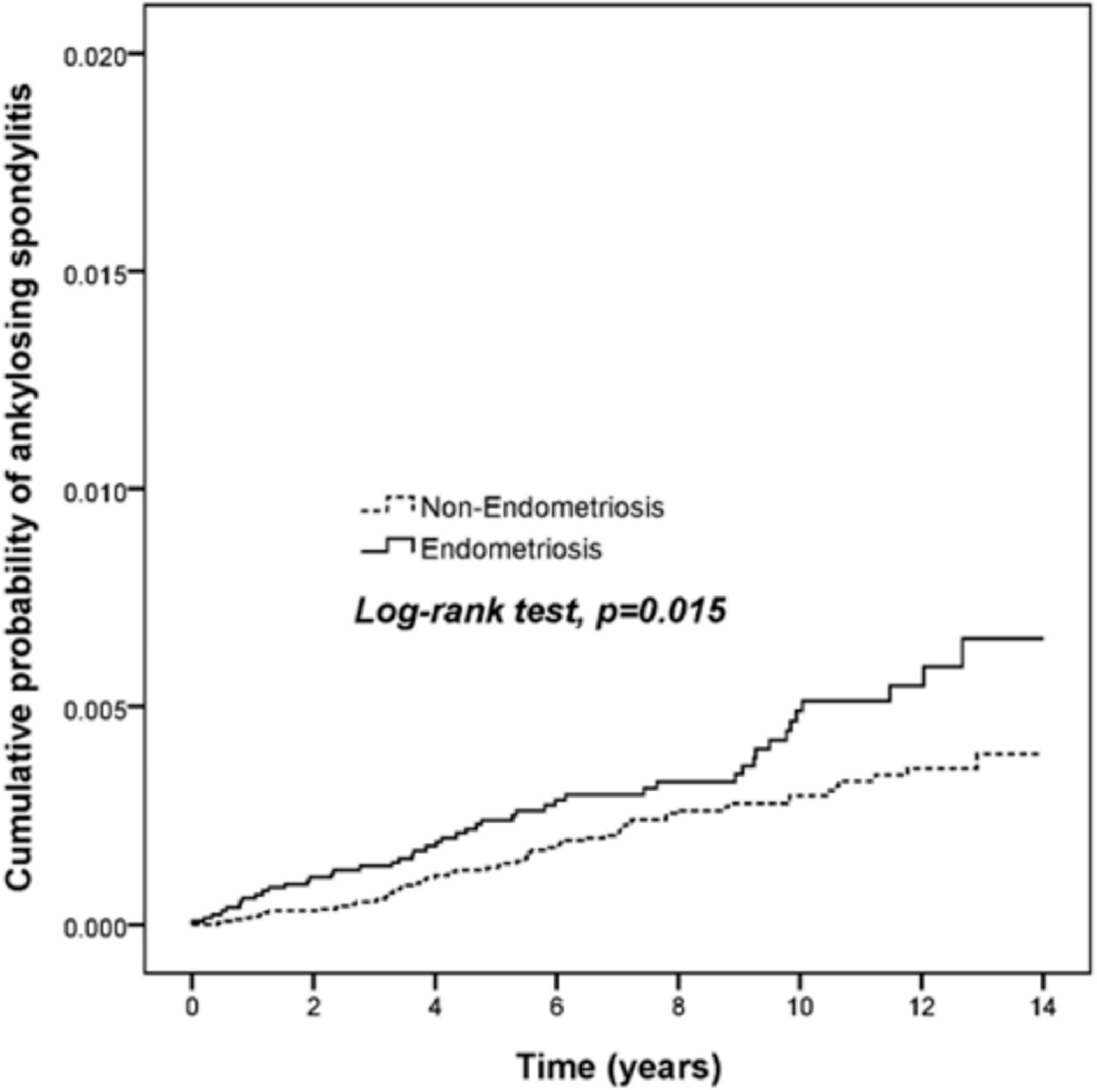
Endometriosis patients display a higher risk for ankylosing spondylitis. The cumulative probability among 13,145 endometriosis patients and 78,870 age and gender-matched comparison cohort was analyzed by log-rank test.

In a 14-year follow-up period, the cumulative incidence of AS in the endometriosis patients was significantly higher than that in those of non-endometriosis subjects (**Figure 2**, *p* = 0.015). The Cox proportional hazard regression analysis is shown in **Table 2**. Patients with endometriosis had an increased risk for subsequent AS compared with the non-endometriosis comparison cohort (HR = 1.61, 95% CI = 1.11 to 2.35, *p* = 0.013).

**Table 2:** Risk assessment of AS with endometriosis, age, other health problems or medications.

	No. of AS^1^	PY^2^	ID^3^	Crude HR^4^	95% CI^5^	p-value	Adjusted HR^6^	95% CI	p-value
Endometriosis									
No	64	218,984	0.3	1			1		
Yes	47	101,709	0.5	**1.59**	**1.09–2.31**	**0.016**	**1.61**	**1.11–2.35**	0.013
Age (years)									
30–39	55	136,868	0.4	1			1		
40–49	50	157,572	0.3	0.79	0.54–1.16	0.230	0.80	0.54–1.18	0.256
≥50	6	26,253	0.2	0.57	0.25–1.33	0.194	0.61	0.26–1.45	0.264
Hypertension	4	17,399	0.2	0.66	0.24–1.78	0.407	0.79	0.27–2.29	0.662
Hyperlipidemia	1	6,436	0.2	0.45	0.06–3.21	0.424	0.68	0.09–5.20	0.712
Chronic liver disease	1	7,325	0.1	0.39	0.05–2.79	0.348	0.43	0.06–3.09	0.400
Major depressive disorder	4	2,603	1.5	4.60	1.69–12.48	0.003	5.05	1.85–13.78	0.002
COPD^7^	3	3,729	0.8	2.37	0.75–7.45	0.141	2.92	0.92–9.30	0.069
Diabetes	1	7,214	0.1	0.40	0.06–2.84	0.357	0.51	0.07–3.88	0.517
Coronary artery disease	1	3,702	0.3	0.78	0.11–5.60	0.806	1.07	0.14–8.16	0.945
Cerebrovascular disease	1	1,570	0.6	1.85	0.26–13.26	0.540	2.32	0.31–17.39	0.414
Corticosteroids	61	213,121	0.3	**0.60**	**0.41–0.88**	**0.008**	**0.52**	**0.35–0.77**	**0.001**
NSAIDs^8^	98	271,075	0.4	1.35	0.76–2.42	0.309	1.76	0.96–3.23	0.066

^1^AS, Ankylosing spondylitis.

^2^PY, Person-Years.

^3^ID, Incidence density (per 1,000 person-years).

^4^HR, Ha0zard Ratio. HR was estimated by univariate Cox proportional hazard model.

^5^CI, Confidence Interval.

^6^Adjusted HR, Adjusted for age, hypertension, hyperlipidemia, chronic liver disease, major depressive disorder, COPD, diabetes, coronary artery disease, cerebrovascular disease, corticosteroids, and NSAIDs. hazard model.

^7^COPD, Chronic obstructive pulmonary disease.

^8^NSAIDs, Non-steroidal anti-inflammatory drugs. The bold values stand for statistical significance.

### Association of AS With Comorbidities and Medications

An increased risk of AS was observed in patients with major depressive disorder (HR = 5.05, 95% CI = 1.85 to 13.78, *p* = 0.002). A trend of increased risk was also found in patients with COPD, although the *p*-value was not significant (HR = 2.92, 95% CI = 0.92 to 9.30, *p* = 0.069). The application of corticosteroids was associated with a lower risk of AS (HR = 0.52, 95% CI = 0.35 to 0.77, *p* = 0.001), whereas the use of NSAIDs showed an increased risk of AS but not significantly (HR = 1.76, 95% CI = 0.96 to 3.23, *p* = 0.066).

### Stratified Analyses

To determine which endometriosis patients were most susceptible to AS, stratified analyses were conducted in the endometriosis group (**Table 3**). Patients with endometriosis and aged between 40 and 50 years had a higher risk of AS compared with non-endometriosis patients (HR = 2.01, 95% CI = 1.15–3.49, *p* = 0.014). In patients without the use of NSAIDs, patients with endometriosis had a significantly higher incidence of AS compared with non-endometriosis patients (HR = 4.57, 95% CI = 1.41–14.84, *p* = 0.011). Moreover, the HR showed a statistical difference between the two groups with or without NSAID usage (*p* for interaction = 0.031).

**Table 3:** Risk assessment of endometriosis or non-endometriosis subjects with age, corticosteroids, or NSAIDs.

	Endometriosis		Non-Endometriosis		HR	95% C	p-value
	N	No. of AS^1^	N	No. of AS^1^			
Age (years)							
30–39	5,395	20	10,784	35	1.23	0.71–2.13	0.461
40–49	6,500	24	13,025	26	**2.01**	**1.15–3.49**	0.014
≥50	1,197	3	2,375	3	2.14	0.43–10.59	0.353
							p for interaction = 0.443
Corticosteroids							
No	5,352	22	10,697	28	1.73	0.99–3.03	0.055
Yes	7,740	25	15,487	36	1.47	0.88–2.44	0.142
							p for interaction = 0.538
NSAIDs^2^							
No	2,949	9	5,875	4	**4.57**	**1.41–14.84**	0.011
Yes	10,143	38	20,309	60	1.35	0.90–2.03	0.143
							**p for interaction = 0.031**

^1^AS, Ankylosing spondylitis.

^2^NSAIDs, non-steroidal anti-inflammatory drugs. The bold values stand for statistical significance.

The association of endometriosis and ankylosing spondylitis was more prominent at ages 40–49 compared with ages 30–39. The exact mechanism of this age-related association is unknown. One possibility is that at a younger stage, the pathogenesis of AS is contributed to by genetic and environmental factors. While in late-onset AS, hormones and chronic inflammation in the uterus may play more important roles.

## Discussion

To our knowledge, this is the first study to use a nationwide population-based database to evaluate the relationship between endometriosis and AS. We found a statistically significant association between endometriosis and the risk of AS (HR = 1.59, 95% CI = 1.09–2.31, *p* = 0.016). Endometriosis patients without the use of NSAIDs were found to have a significantly higher incidence of AS compared with NSAID users (HR = 4.57, 95% CI = 1.41–14.84, *p* = 0.011). As such, we infer that endometriosis is probably an independent risk factor for AS.

The underlying mechanism between endometriosis and AS with an increased comorbidity is unclear. We hypothesized several possibilities to explain the mechanism. First, endometriosis and AS are probably related to gene susceptibility. The Killer immunoglobulin-like two-domain short-tail receptor (KIR2DS5) is a key receptor of NK cells that promotes an early immune response to infection. Previous studies indicate that the KIR2DS5 gene is a protective factor against endometriosis and AS (4–6). In a special case, a woman with endometriosis was diagnosed with 13 co-morbidities, including SLE and AS (7). She was found homozygous for rs2476601 SNP of the *PTPN22* gene, and heterozygous for both rs27434 and rs30187 SNPs of the *ERAP1* gene, which have been proven to be related to AS (8–10). Secondly, both endometriosis and AS are involved in inflammatory responses induced by inflammatory mediators (11–13). For endometriosis, the vigorous inflammatory response will cause endometrial cells to release more chemokines, namely, IL-1β, IL-6, IL-8, TNF-α, and IL-17 (10, 11). Also, increased levels of IL-6, TNF-α, IL-1β, and IL-17 could be detected in AS patients (12, 14, 15). Moreover, both TNF-α and IL-17 inhibitors have therapeutic effects on AS (16, 17). Some studies also suggested that anti-TNF-α or TNF-inhibitors can significantly inhibit the progression of endometriosis lesions (18–20).

Patients with major depressive disorder were also associated with an increased risk of AS, with evidence suggesting that inflammatory cytokines are involved in the pathogenesis of major depression (21). Hypotheses such as ‘macrophage theory of depression’ and the ‘cytokine hypothesis of depression’ were proposed to describe a higher expression of IL-1, TNF‐α, IL-6, and IL-8 in patients suffering from depression (22). The balance between pro- and anti-inflammatory cytokines is supposed to be altered in subgroups of depressed patients, resulting in symptoms seen in comorbid depression associated with inflammation such as ankylosing spondylitis. Cytokine blockers may be used to improve clinical outcomes in major depressive disorders (23). Several studies also suggested that AS increases the risk of depressive disorders (24–26). Therefore, major depressive disorder and AS may interact with each other due to immune dysfunction.

Patients with COPD might show a higher risk of AS, with the p-value being around 0.05 (HR = 2.92, 95% CI = 0.92 to 9.30, *p* = 0.069). Two studies have demonstrated an increased incidence of COPD in patients with AS (27, 28). Our previous study showed that COPD was associated with a higher risk of SLE (HR = 1.75, 95% CI = 1.06 to 2.89, *p* = 0.028) (29). COPD patients could produce autoantibodies that were reactive to those antigens that are also found in many autoimmune diseases (30). All these findings indicate that autoimmunity may be linked to COPD and AS. The effect of smoking on both COPD and AS should be considered, but such information is excluded from the database.

Corticosteroids are associated with many complications, such as osteoporosis and subsequent bone fractures, hypertension, and neuropsychiatric disorders (31). However, our data showed that the cumulative daily dose of corticosteroids for more than 30 days has a lower risk of developing AS (HR = 0.52, 95% CI = 0.35 to 0.77, *p* = 0.001). This is the first study to display the protective effects of corticosteroids on endometriosis patients against the risks of AS. The exact mechanism remains unknown, perhaps because of its anti-inflammatory effects. In comparison, the use of NSAIDs seemed to increase the risk of AS, though without statistical significance (HR = 1.76, 95% CI = 0.96 to 3.23, *p* = 0.066). Therefore, clinicians are advised to assess the medication effect on AS risk during follow-up of endometriosis patients.

In our study, we also found the association of endometriosis and ankylosing spondylitis to be more prominent in the group aged 40–49 compared with individuals aged 30–39. The exact mechanism of this age-associated memory is still unknown. One possibility is that at a younger stage, the pathogenesis of AS is contributed to by genetic and environmental factors. While in late-onset AS, hormones and chronic inflammation in the uterus may play more important roles. Further studies are needed to elucidate the exact mechanistic causes and the correlation between age and disease onset.

There were some limitations to our study. Firstly, endometriosis might be associated with multifactorial factors such as BMI, alcohol use, smoking, family history, and menstrual status, which were not available in our database. Nevertheless, we matched alcohol and smoking-related diseases, including COPD, liver disease, and diabetes, to minimize these limitations. Secondly, laboratory data such as hormone levels and inflammatory markers were not available in this claim-based database. Moreover, the severity of endometriosis was not recorded in this database, but we had matched both groups on steroid and NSAID usage and did stratified analyses to reduce this bias.

However, the strength of our study is the population-based data based on the whole Taiwanese population, which provided enough sample size to minimize selection bias. Furthermore, the follow-up time of this work was 14 years, enabling us to observe the subsequent occurrence of AS, yet further studies are needed to support our findings.

## Conclusion

In this retrospective population-based cohort study, we found a potentially higher risk of having AS in patients with endometriosis. NSAID use might reduce the risk of developing AS. Clinicians should pay attention to the risk of AS in patients with endometriosis and possible underlying endometriosis in managing AS patients. Further research may help clarify the possible mechanisms between endometriosis and AS.

## Data Availability Statement

The data analyzed in this study is subject to the following licenses/restrictions: The data in this article is provided by and used under the permission of the Bureau of National Health Insurance. Data would be shared with permission of the Bureau of National Health Insurance. Requests to access these datasets should be directed to James Cheng-Chung Wei, jccwei@gmail.com.

## Ethics Statement

This study was approved by the Chung Shan Medical University Hospital (IRB, CS19009).

## Author Contributions

ZHY, JCCW and ZZY accessed the data and supervised the project. H-YL, K-SH, YZ and YHW performed bioinformatics analysis and wrote the manuscript. BSC wrote the manuscript and revised the literature. All authors contributed greatly to the production and submission of the work.

## Funding

This work was supported by the Sanming Project of Medicine in Shenzhen (grant number SZSM201602087), the National Natural Science Foundation of China (grant number 81102266), Medical Scientific Research Foundation of Guangdong Province (grant number A2018089), the Shenzhen Science and Technology Project (grant number JCYJ20180504170414637), and the Shenzhen Futian Public Welfare Scientific Research Project (grant numbers FTWS2021006).

## Author Disclaimer

Researchers were independent of the funders. All authors had full access to all of the data (including statistical reports and tables) in the study and can take responsibility for the integrity of the data and the accuracy of the data analysis.

## Conflict of Interest

The authors declare that the research was conducted in the absence of any commercial or financial relationships that could be construed as a potential conflict of interest.

## Publisher’s Note

All claims expressed in this article are solely those of the authors and do not necessarily represent those of their affiliated organizations, or those of the publisher, the editors and the reviewers. Any product that may be evaluated in this article, or claim that may be made by its manufacturer, is not guaranteed or endorsed by the publisher.

## References

[1] Giudice LC Kao LC. Endometriosis. Lancet (2004) 364(9447):1789–99. doi: 10.1016/S0140-6736(04)17403-5 15541453

[2] García-Gómez E Vázquez-Martínez ER Reyes-Mayoral C Cruz-Orozco OP Camacho-Arroyo I Cerbón M. Regulation of Inflammation Pathways and Inflammasome by Sex Steroid Hormones in Endometriosis. Front Endocrinol (Lausanne) (2019) 10:935. doi: 10.3389/fendo.2019.00935 32063886 PMC7000463

[3] Shigesi N Kvaskoff M Kirtley S Feng Q Fang H Knight JC. The Association Between Endometriosis and Autoimmune Diseases: A Systematic Review and Meta-Analysis. Hum Reprod Update (2019) 25(4):486–503. doi: 10.1093/humupd/dmz014 31260048 PMC6601386

[4] Nowak I Płoski R Barcz E Dziunycz P Kamiński P Kostrzewa G. KIR2DS5 in the Presence of HLA-C C2 Protects Against Endometriosis. Immunogenetics (2015) 67(4):203–9. doi: 10.1007/s00251-015-0828-3 PMC4357646 25724317

[5] Nowak I Majorczyk E Wiśniewski A Pawlik A Magott-Procelewska M Passowicz-Muszyńska E. Does the KIR2DS5 Gene Protect From Some Human Diseases? PLoS One (2010) 5(8):e12381. doi: 10.1371/journal.pone.0012381 20865034 PMC2928722

[6] Rezaei R Mostafaei S Aslani S Jamshidi A Mahmoudi M. Association Study Between Killer Immunoglobulin-Like Receptor Polymorphisms and Ankylosing Spondylitis Disease: An Updated Meta-Analysis. Int J Rheum Dis (2018) 21(10):1746–55. doi: 10.1111/1756-185X.13408 30398028

[7] Matalliotaki C Matalliotakis M Zervou MI Trivli A Matalliotakis I Mavromatidis G. Co-Existence of Endometriosis With 13 non-Gynecological Co-Morbidities: Mutation Analysis by Whole Exome Sequencing. Mol Med Rep (2018) 18(6):5053–7. doi: 10.3892/mmr.2018.9521 PMC6236265 30272298

[8] Tizaoui K Kim SH Jeong GH Kronbichler A Lee KS Lee KH. Association of PTPN22 1858c/T Polymorphism With Autoimmune Diseases: A Systematic Review and Bayesian Approach. J Clin Med (2019) 8(3):347. doi: 10.3390/jcm8030347 PMC6462981 30871019

[9] Evans DM Spencer CC Pointon JJ Su Z Harvey D Kochan G. Interaction Between ERAP1 and HLA-B27 in Ankylosing Spondylitis Implicates Peptide Handling in the Mechanism for HLA-B27 in Disease Susceptibility. Nat Genet (2011) 43(8):761–7. doi: 10.1038/ng.873 PMC3640413 21743469

[10] Cortes A Pulit SL Leo PJ Pointon JJ Robinson PC Weisman MH. Major Histocompatibility Complex Associations of Ankylosing Spondylitis are Complex and Involve Further Epistasis With ERAP1. Nat Commun (2015) 6:7146. doi: 10.1038/ncomms8146 25994336 PMC4443427

[11] Yang H Chen Y Xu W Shao M Deng J Xu S. Epigenetics of Ankylosing Spondylitis: Recent Developments. Int J Rheum Dis (2021) 24(4):487–93. doi: 10.1111/1756-185X.14080 33608999

[12] Kong W Tang Y Tang K Yan Z Liu T Tao Q. Leukemia Inhibitory Factor is Dysregulated in Ankylosing Spondylitis and Contributes to Bone Formation. Int J Rheum Dis (2022) 25(5):592–600. doi: 10.1111/1756-185X.14312 35238474

[13] Feng HY Chan CH Chu YC Qu XM Wang YH Wei JC. Patients With Ankylosing Spondylitis Have High Risk of Irritable Bowel Syndrome: A Long-Term Nationwide Population-Based Cohort Study. Postgrad Med (2022) 134(3):290–6. doi: 10.1080/00325481.2022.2041338 35139724

[14] Londono J Romero-Sanchez MC Torres VG Bautista WA Fernandez DJ Quiroga Jde A. The Association Between Serum Levels of Potential Biomarkers With the Presence of Factors Related to the Clinical Activity and Poor Prognosis in Spondyloarthritis. Rev Bras Reumatol (2012) 52(4):536–44. doi: 10.1590/S0482-50042012000400006 22885421

[15] Raychaudhuri SP Raychaudhuri SK. Mechanistic Rationales for Targeting Interleukin-17A in Spondyloarthritis. Arthritis Res Ther (2017) 19(1):51. doi: 10.1186/s13075-017-1249-5 28270233 PMC5341175

[16] Maxwell LJ Zochling J Boonen A Singh JA Veras MM Tanjong Ghogomu E. TNF-Alpha Inhibitors for Ankylosing Spondylitis. Cochrane Database Syst Rev (2015) 4(4):Cd005468. doi: 10.1002/14651858.CD005468.pub2 PMC11200207 25887212

[17] Pavelka K Kivitz A Dokoupilova E Blanco R Maradiaga M Tahir H. Efficacy, Safety, and Tolerability of Secukinumab in Patients With Active Ankylosing Spondylitis: A Randomized, Double-Blind Phase 3 Study, MEASURE 3. Arthritis Res Ther (2017) 19(1):285. doi: 10.1186/s13075-017-1490-y 29273067 PMC5741872

[18] Kondo W dal Lago EA Noronha L Olandoski M Kotze PG Amaral VF. Effect of Anti-TNF-α on Peritoneal Endometrial Implants of Rats. Rev Col Bras Cir (2011) 38(4):266–73. doi: 10.1590/S0100-69912011000400011 21971861

[19] Liu Y Sun L Hou Z Mao Y Cui Y Liu J. rhTNFR: Fc Suppresses the Development of Endometriosis in a Mouse Model by Downregulating Cell Proliferation and Invasiveness. Reprod Sci (2016) 23(7):847–57. doi: 10.1177/1933719115620495 26674323

[20] Ajrawat P Touma Z Sari I Taheri C Diaz Martinez JP Haroon N. Effect of TNF-Inhibitor Therapy on Spinal Structural Progression in Ankylosing Spondylitis Patients: A Systematic Review and Meta-Analysis. Int J Rheum Dis (2020) 23(6):728–43. doi: 10.1111/1756-185X.13829 32419337

[21] Varan Ö Babaoğlu H Göker B. Associations Between Depressive Disorders and Inflammatory Rheumatic Diseases. Curr Top Med Chem (2018) 18(16):1395–401. doi: 10.2174/1568026618666180516100805 29766809

[22] Buras A Waszkiewicz N Szulc A Sierakowski S. [Monocytic Parameters in Patients With Rheumatologic Diseases Reflect Intensity of Depressive Disorder]. Pol Merkur Lekarski (2012) 33(198):325–9. doi: 10.5604/17322693.1196386 23437701

[23] Shariq AS Brietzke E Rosenblat JD Barendra V Pan Z McIntyre RS. Targeting Cytokines in Reduction of Depressive Symptoms: A Comprehensive Review. Prog Neuropsychopharmacol Biol Psychiatry (2018) 83:86–91. doi: 10.1016/j.pnpbp.2018.01.003 29309829

[24] Shen CC Hu LY Yang AC Kuo BI Chiang YY Tsai SJ. Risk of Psychiatric Disorders Following Ankylosing Spondylitis: A Nationwide Population-Based Retrospective Cohort Study. J Rheumatol (2016) 43(3):625–31. doi: 10.3899/jrheum.150388 26834219

[25] Hakkou J Rostom S Mengat M Aissaoui N Bahiri R Hajjaj-Hassouni N. Sleep Disturbance in Moroccan Patients With Ankylosing Spondylitis: Prevalence and Relationships With Disease-Specific Variables, Psychological Status and Quality of Life. Rheumatol Int (2013) 33(2):285–90. doi: 10.1007/s00296-012-2376-6 22441961

[26] Ben Tekaya A Mahmoud I Hamdı I Hechmı S Saıdane O Tekaya R. [Depression and Anxiety in Spondyloarthritis: Prevalence and Relationship With Clinical Parameters and Self-Reported Outcome Measures]. Turk Psikiyatri Derg (2019) 30(2):90–8. 31487374

[27] Hemminki K Liu X Ji J Sundquist K Sundquist J. Subsequent COPD and Lung Cancer in Patients With Autoimmune Disease. Eur Respir J (2011) 37(2):463–5. doi: 10.1183/09031936.00070410 21282811

[28] Lambert J Chekroun M Gilet H Acquadro C Arnould B. Assessing Patients' Acceptance of Their Medication to Reveal Unmet Needs: Results From a Large Multi-Diseases Study Using a Patient Online Community. Health Qual Life Outcomes (2018) 16(1):134. doi: 10.1186/s12955-018-0962-3 29976222 PMC6034222

[29] Shi LH Huang JY Liu YZ Chiou JY Wu R Wei JC. Risk of Systemic Lupus Erythematosus in Patients With Human Papillomavirus Infection: A Population-Based Retrospective Cohort Study. Lupus (2018) 27(14):2279–83. doi: 10.1177/0961203318809179 30451639

[30] Packard TA Li QZ Cosgrove GP Bowler RP Cambier JC. COPD is Associated With Production of Autoantibodies to a Broad Spectrum of Self-Antigens, Correlative With Disease Phenotype. Immunol Res (2013) 55(1-3):48–57. doi: 10.1007/s12026-012-8347-x 22941590 PMC3919062

[31] McDonough AK Curtis JR Saag KG. The Epidemiology of Glucocorticoid-Associated Adverse Events. Curr Opin Rheumatol (2008) 20(2):131–7. doi: 10.1097/BOR.0b013e3282f51031 18349741

